# Exhausted but Not Senescent T Lymphocytes Predominate in Lupus Nephritis Patients

**DOI:** 10.3390/ijms232213928

**Published:** 2022-11-11

**Authors:** Georgios Lioulios, Zoi Mitsoglou, Asimina Fylaktou, Aliki Xochelli, Michalis Christodoulou, Stamatia Stai, Eleni Moysidou, Afroditi Konstantouli, Vasiliki Nikolaidou, Aikaterini Papagianni, Maria Stangou

**Affiliations:** 1 Department of Nephrology, School of Medicine, Aristotle University of Thessaloniki, General Hospital “Hippokratio”, 54642 Thessaloniki, Greece; 2 Department of Immunology, National Peripheral Histocompatibility Center, General Hospital “Hippokratio”, 54642 Thessaloniki, Greece

**Keywords:** lupus nephritis, immune senescence, immune exhaustion, histology, SLEDAI score, T lymphocytes

## Abstract

Lupus nephritis (LN), a chronic inflammatory disease, is characterized by the substantial disruption of immune homeostasis. This study examines its effects on the T lymphocyte phenotype and, particularly, its senescence- and exhaustion-related immune alterations. T cell subpopulations were determined with flow cytometry in 30 LN patients and 20 healthy controls (HCs), according to the expression of senescence- (CD45RA, CCR7, CD31, CD28, CD57), and exhaustion- (PD1) related markers. The immune phenotype was associated with disease activity and renal histology. LN patients were characterized by pronounced lymphopenia, mainly affecting the CD4 compartment, with a concurrent reduction in the naïve, central and effector memory subsets compared to the HCs. In the CD8 compartment, the naïve subsets were significantly lower than that of the HCs, but a shift in the T cells occurred towards the central memory population. CD4+PD1+ and CD8+PD1+ cells were increased in the LN patients compared to the HCs. However, in CD4 T cells, the increase was limited to CD45RA+, whereas in CD8 T cells, both CD45RA+ and CD45RA− subsets were affected. Disease activity was correlated with CD4+PD1+ and highly differentiated CD4+CD28-CD57+ cells. Histology was only associated with CD4 T cell disturbances, with stage IV presenting reduced naïve and increased senescent subsets. Exhausted T lymphocyte subpopulations predominate within LN patients, while the T cell phenotype varies depending on disease activity.

## 1. Introduction

Immunosenescent phenotype deviations constitute a recently described global phenomenon, which includes the characteristic structural alterations of immune cells, happening as a consequence to advanced ageing. Nevertheless, this is also accompanied by the secondary change and conformation of their functional activity [1,2,3]. Apart from ageing, chronic inflammatory diseases and conditions have been implicated in the early development and progression of this phenomenon as chronic exposure to antigens or pathogens may exacerbate cell activation. However, they may simultaneously stimulate adaptive mechanisms in order to regulate immune-mediated reactions and control inflammation [4,5,6].

In addition to the immunosenescence procedure, the exhaustion of the immune system constitutes another complication usually seen in the presence of chronic antigenic stimulation [4,7]. The definition of “exhausted” T cells relies on three parameters, which are necessary for the definition of T lymphocytes as exhausted cells: cellular dysfunction (absence of expected effector response to exogenous stimulation); enduring exposure to a certain stimulus; and the expression of particular surface receptors, such as programmed cell-death protein 1 (PD1) [8]. There are several reports in the literature which support the notion that PD1 expression on T lymphocytes does not necessarily represent exhausted cells but, rather, it can be expressed on T cells as a designation of their activation. Moreover, in healthy individuals, PD1 was predominantly expressed by effector memory (EM) T cells rather than terminally differentiated effector memory CD45RA+ (EMRA) T cells, and it was never expressed on naïve T cells [7,9,10]. Therefore, in the present study, we examined PD1 expression on both CD45RA− and CD45RA+ T cells in order to determine exhaustion in T cells of different differentiation statuses.

It is essential to discriminate immune-exhaustion from immune-senescence. T lymphocytes are the cells usually involved in this procedure. Phenotypic differences rely on the presence of PD1 on the exhausted cells, while the different expression of CD45RA, CD57 and CD28 molecules denote alterations of senescent cells. Exhausted cells are not anergic; they can be stimulated but not differentiated properly. Nevertheless, senescent T cells have “deleterious” and cytotoxic effects [11].

We have previously estimated phenotypic alterations of T and B lymphocytes in patients with end-stage renal disease (ESRD), at pre-dialysis stage, on dialysis treatment, and after renal transplantation [12,13,14,15]. The effect of ESRD on the immune phenotype proved to be unique; similar to senescence, albeit with substantial differences [12]. Based on these previous findings, we decided to expand the research on the immunosenescence and immune exhaustion phenomena to systemic lupus erythematosus (SLE), a disease comprehensively acknowledged as sustained chronic inflammation. Kidney involvement in SLE is regarded as a major organ complication, resulting as a consequence of systemic-cell-mediated mechanisms, encompassing mainly dendritic cells and activated lymphocytes [16,17,18]. The presence of kidney involvement in SLE determines disease morbidity and mortality [16,19]. In the present study, we recruited SLE patients with biopsy-proven lupus nephritis (LN) to investigate the effect of chronic systemic inflammation with major organ complications in these two phenomena. We evaluated the presence of specific surface molecules on T lymphocytes, which can differentiate them in naïve, senescent, and exhausted subpopulations in LN patients, and we compared the findings with healthy controls of similar sex, ethnicity, and age.

## 2. Results

### 2.1. Characteristics of patients

Clinical and histological data at the time of diagnosis and the time of evaluation are described in Table 1. The mean age of the LN patients at the time of evaluation was 43 ± 16 years, eGFR was 98 ± 25 mL/min/1.73 m^2^, was total urine protein (Uprot) was 1.5 ± 0.7 g/24 h. All patients had microscopic hematuria and proteinuria > 500 mg/24 h.

At the time of evaluation, 27/30 (90%) patients were under steroid treatment, 3/30 (10%) were on calcineurin inhibitors, and 30/30 (100%) were on Mycophenolate mofetil (MMF) and Hydroxychloroquine.

### 2.2. Evaluation of the Immune Phenotype

#### 2.2.1. Differences in T Lymphocyte Phenotype between LN Patients and HCs

**Figure 1:**
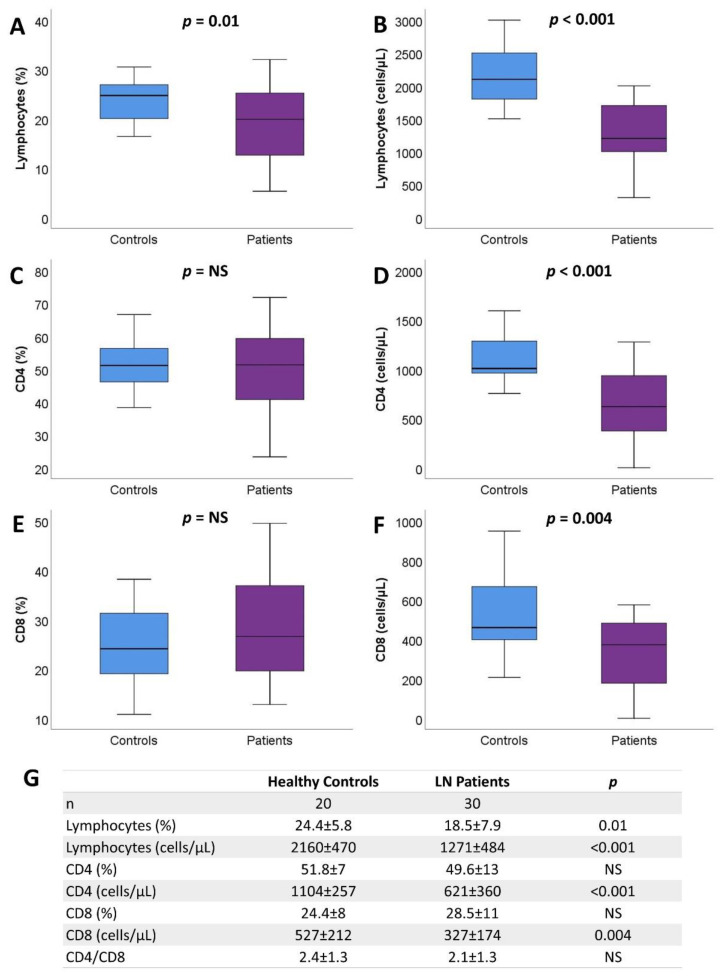
Differences in percentage (**A**,**C**,**E**) and absolute number (**B**,**D**,**F**) of total lymphocytes, CD4+, and CD8+ between healthy controls and LN patients. The mean ± SD of total lymphocytes and CD4 and CD8 subpopulations are described (**G**) (NS: non-significant, LN: lupus nephritis).

The percentage and absolute number of total lymphocytes were significantly reduced in patients with LN compared to the healthy controls. The reduction affected both the CD4 and CD8 subpopulations, although elimination was more prominent among the CD4 compartment, leading to a slightly reduced CD4/CD8 ratio (Figure 1).

#### 2.2.2. Differences in CD4 and CD8 Lymphocyte Subpopulations between LN Patients and Healthy Controls, Based on the Presence of Senescent Markers

Total CD4+ lymphocytes were reduced in the LN patients; however, this reduction specifically affected some of the naïve and memory CD4 subtypes. Subpopulations that were significantly reduced in the LN patients were CD4+CD45RA+CCR7+ (naïve), CD4+CD45RA−CCR7+ (Central Memory, CM), CD4+CD45RA−CCR7− (EM), and CD4+CD45RA+CCR7− (EMRA) cells. CD4+CD45RA+CD31+ recent thymic emigrants (RTEs) were not significantly affected as is depicted in Table 2, a finding which indicates that the thymus function remained intact. Moreover, CD4+CD28− T cells, including CD28-EMRA and CD4+CD28−CD57+ T cells, which are of the most senescent subsets, remained unaltered between the two cohorts.

Regarding CD8 lymphocyte subpopulations (Table 3), changes were more prominent as most naïve subpopulations were significantly reduced, including CD8+CD31+ (RTEs), CD8+CD45RA+CCR7+ (naïve), CD8+CD45RA+CD57−, CD8+CD28+CD57− T cells. However, very interestingly, “advanced differentiated” CD8 cells were also reduced, including those cells lacking the CD28 molecule, namely CD8+CD45RA+CCR7− (ΕΜRA), CD8+CD45RA+CCR7−CD28− (CD28− EMRA), and CD8+CD45RA+CD57+. The CD8 compartment shifted towards CD8+CD45RA−CCR7+ (CM) T cells, which were significantly increased in LN patients compared to the healthy controls.

**Figure 2:**
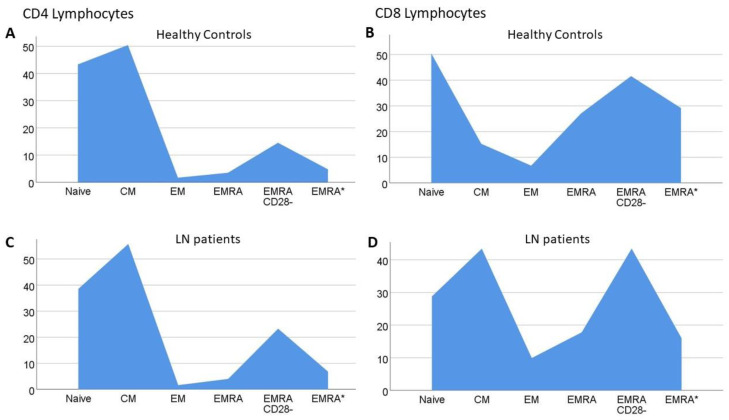
Percentage distribution of CD4 (**A**,**C**) and CD8 (**B**,**D**) lymphocyte subsets in healthy controls and lupus nephritis (LN) patients, respectively. CM: central memory; EM: effector memory; EMRA: effector memory re-expressing CD45RA; EMRA*: effector memory re-expressing CD45RA, defined as CD45RA+CD28−.

Figure 2 depicts the differences in the proportion of certain lymphocyte types between the LN patients and healthy controls (HC). No remarkable changes in the frequency of CD4 subtypes in the LN patients was noticed, and although there was a slight increase in the percentage of CM and CD45RA+CCR7−CD28− cells, these differences did not reach statistical significance. However, a completely different shape was evident regarding CD8 fluctuations. A reduction in the proportion of CD45RA+CCR7+ (naïve) cells was prominent, accompanied with a similar reduction in the most advanced differentiated cell type, namely CD45RA+CD28−, and a concomitant significant increase in the percentage of CD45RA-CCR7+ (CM) cells were the main changes in the CD8 compartment.

#### 2.2.3. Differences in the Expression of PD1 Molecule on CD4 and CD8 Lymphocytes, between LN Patients and Healthy Controls

**Figure 3:**
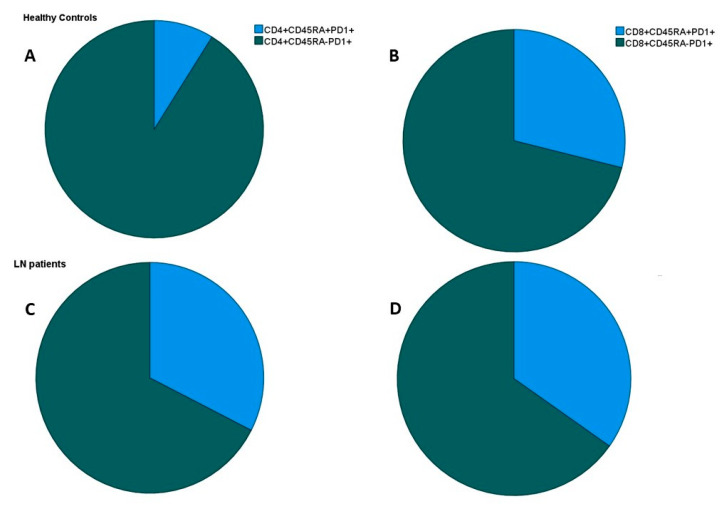
Ratio of CD4+CD45RA+PD1+/CD4+CD45RA−PD1+ and CD8+CD45RA+PD1+/CD8+CD45RA−PD1+ cells in healthy controls (**A**,**B**) and lupus nephritis (LN) patients (**C**,**D**).

Differences in the expression of marker PD1 on CD4 and CD8 lymphocytes between the LN patients and HCs are listed on Table 4. A statistically significant increased expression of the PD1 molecule was noticed within the LN patients, affecting both CD4 and CD8 cells. Further analysis of the PD1 expression on CD45RA+ and CD45RA− lymphocytes revealed that the increase in PD1 expression on CD4 cells mainly affected CD4+CD45RA+ cells, while within the CD8 compartment, PD1 expression was similar between the CD8+CD45RA+ and CD8+CD45RA− subpopulations. (Figure 3). The ratio of CD4 exhausted/active cells (CD4+CD45RA+PD1+/CD4+CD45RA−PD1+) was significantly increased in the LN patients versus the HCs, 0.34 ± 0.3 versus 0.11 ± 0.1, respectively, *p* = 0.004, while the ratio in CD8 exhausted/active cells (CD8+CD45RA+PD1+/CD8+CD45RA−PD1+) was similar between the LN patients and HCs, 0.69 ± 0.7 vs. 1.2 ± 1.5, *p* = 0.2.

### 2.3. Correlation of Lymphocyte Phenotype with Clinical and Laboratory Findings

#### 2.3.1. Correlation with Disease Activity

The SLEDAI-2K index showed a positive correlation with the whole number of CD4+PD1+, r = 0.6, *p* = 0.01, as well as the subgroups CD4+CD45RA+PD1+, r = 0.5, *p* = 0.03, and CD4+CD45RA−PD1+, r = 0.5, *p* = 0.02, but not with CD8+PD1+ cells. Furthermore, the SLEDAI-2K index was found to show a negative correlation with the number of CD4+CD28−CD57+, r = −0.5, *p* = 0.03.

Of the remaining laboratory findings, the SLEDAI-2K index showed a negative correlation with C3 levels, r = −0.5, *p* = 0.02; with serum albumin levels, r = −0.5, *p* = 0.04; and a positive correlation with serum creatinine levels, r = 0.6, *p* = 0.01.

#### 2.3.2. Correlation with Histological Findings

The differentiation of the lymphocyte phenotype according to histological classification mainly regarded the CD4 lymphocytes and their subtypes, as patients with stage IV LN, to have a significant reduction in CD4 lymphocytes, mainly elaborating naïve CD4 and CD4+CD31+ cells. On the contrary, there was an increase in CM CD4 lymphocytes, as well as advanced differentiated cells, such as CD4+CD28− and CD4+CD45RA+CCR7−CD28− cells. Similar results were not described for the CD8 compartment (Table 5).

## 3. Discussion

In the present study, we investigated the phenotypic changes of T lymphocytes in patients with lupus nephritis. We first noticed that the fluctuation of the CD4 and CD8 lymphocyte subpopulations followed a different course in the LN patients. CD4 lymphocytes were significantly reduced in the LN patients; however, the distribution between CD45RA+CCR7+/CD45RA−CCR7+/CD45RA−CCR7−/CD45RA+CCR7 remained similar. A small reduction in CD45RA+CCR7+ cells was accompanied by an increase in central memory (CD45RA−CCR7+) and advanced differentiated CD4+CD45RA+CCR7−CD28− cells.

CD8 lymphocytes, on the other hand, showed more substantial changes, namely, reduced naïve (CD45RA+CCR7+) cells, but also a simultaneous and significant reduction in the advanced differentiated CD45RA+CD28− cells, defined as “senescent” lymphocytes, but these changes were followed by a significant increase in CM CD8 cells.

CM cells(CD45RA−CCR7+), have the ability to migrate to areas of secondary lymphoid organs through high endothelial venules [1,20]. These cells are more sensitive than naïve T lymphocytes and are rapidly stimulated by antigens, assuming the adequate activation of B lymphocytes and dendritic cells [20]. Our results regarding the increased CM cells in the LN patients are in accordance with previous ones, showing an accumulation of certain T lymphocyte subsets in active SLE. The predominance of CD45RO expression (which replaces the CD45RA isoform after naïve T cell activation [1]) has been described during SLE activation, during which its levels have a significant positive correlation with time from activation. On the other hand, EM CD8 cells have been found to migrate from peripheral blood to the kidney, and to be excreted in the urine of patients with active LN [21,22] At the same time, the CD28 receptor is gradually lost from T lymphocytes, leading to CD28null, highly atherogenic highly differentiated cells [23,24]. In our results, we did not find a significant elimination of the CD28 receptor in either CD4 or CD8 cells. Instead, lymphocytes lacking CD28 surface molecule, as well as CD57+ lymphocytes, both being advanced differentiated cells, were reduced. There was a clear shift to memory cells, particularly CM cells, and this was more prominent in the CD8 compartment.

Histology classification in our LN patients was mainly correlated with changes of CD4 lymphocytes, while CD8 cells did not seem to play a significant role in renal pathology. Patients with active and proliferative lesions, classified as class III/IV, had reduced peripheral CD4 cells, characterized by a tendency to move from naïve towards ageing CD4 cell types. In particular, a significant reduction in RTE (CD4+CD31+) T cells, together with increased CM and CD4+CD28− cells, especially the CD4+CD28−CD57+ and CD4+CD45RA+CCR7−CD28− cells, were the main characteristics of stage III/IV LN patients’ immune phenotype. The close association between LN classification and specific CD4 lymphocytes can be explained in several different ways. The proliferative types of LN indicate a more active disease with innovative immunological deviations, including the presence of autoantibodies, lymphocyte stimulation, and complement activation; changes that can justify the specific alterations of the CD4 phenotype we described here. Renal function impairment, usually accompanied with active LN lesions, may also contribute to T lymphocyte disturbances, as we have described before. Additionally, immunosuppressive treatment, per se, is potentially accompanied by detrimental effects on the immune phenotype. Recent evidence, however, describes that immunosuppressive treatment in active LN had no impact on specific CD4 subtypes, especially CD4+CD28null and CD4-activated cells [25]. Additionally, as our patients were currently all had the same immunosuppression, we anticipate that treatment effect is limited by the variability of their immune profile.

The most important finding in our study was the increased expression of PD1 on both CD4 and CD8 T lymphocytes. Moreover, while CD8 T lymphocytes expressing PD1 were both EMRA, CD8+CD45RA+PD1+, and memory, CD8+CD45RA−PD1+, in the CD4 compartment expression of PD1 was more prominent on the EMRA CD4+CD45RA+PD1+ T cells, which were increased compared to the controls. The memory CD4+CD45RA−PD1+ T cells were on the same level in both cohorts.

Programmed cell-death protein 1 (PD1 or CD279) is the checkpoint immunoinhibitory receptor of the activated T lymphocytes. It consists of a 55kDa transmembrane protein, usually expressed on the surface of T lymphocytes following antigen-mediated activation through the T cell receptor (TCR). Therefore, PD1 is not expressed on naïve T cells, but only on effector T cells and on tolerant, regulatory, memory, and follicular helper T cells. Although it is classically regarded as a marker of exhaustion, PD1 expression is upregulated in chronic infection after sustained exposure to a specific antigen, even when the responsible antigen has been cleared. Recent research has shown evidence of a close association between PD1 expression and increased T cell metabolic activity [7,10,11,26].

The role of PD1 expression in SLE patients has recently gained great interest; however, the results are controversial. Most investigators support a destructive effect of PD1 expression in LN as the frequency of CD4+PD1+ and CD4+CXCR5−PD1+ peripheral T cells has been correlated with increased levels of INF-gamma and IL-17, as well as with disease activity [27,28]. Meanwhile, others have shown that the PD1/PDL1 ratio is reduced in active LN, with vitamin D playing central role in the inflammatory immune process [29]. Moreover, exhausted CD8+PD1+ T cells exert a reduced cytotoxic function and a reduced capacity of IL-2, IFN-γ, and TNF production [30]. This reduced effector capacity of CD8 T cells might contribute to autoimmunity through the reduced regulation of autoreactive B cells [31]. Thus, it might evolve into SLE pathogenesis. Experimental models have proved the detrimental effect of PD1 expression on T lymphocytes in several mice models of LN. A blockade of PD1, by using antiPD1mAb, can result in a reduction in IFN-γ, IL-17, IL-10, and anti-dsDNA autoantibodies. It can also eliminate the urinary excretion of activated T cells, and thus ameliorate disease activity and reduce mortality in NZB/W F1 mice. On the other hand, blocking the ligand of PD1 leads to increased peripheral CD4+PD1+ T cells and accelerates lupus nephritis [32,33]. The regulation of PD1 expression in BWF(1) mice is a prerequisite of immune tolerance, mediated by CD8+Foxp3+ T cells [34]. However, almost all studies, both human and experimental, have been dedicated to exploring the role of PD1 and the PD1-PDL1 axis on CD4 lymphocytes, and no results are available so far regarding CD8+PD1+ cells. Our study is the first one to prove the important role of PD1 expression on CD8 lymphocytes in LN. More interestingly, when we distinguished exhausted EMRA (CD45RA+PD1+) from memory (CD45RA−PD1+) cells, we found that, in the CD4 compartment, the main population that increased in LN was EMRA cells, while in the CD8 compartment, both cell subsets increased equally.

This study has several limitations. A major limitation is the relatively small number of patients and healthy controls included. Moreover, we did not evaluate the patients’ phenotype upon SLE diagnosis; hence, all individuals were on immunosuppressive treatment at the time of inclusion. Immunosuppression possibly affects the phenotype of T cells, but its effects on T cells’ function might be dose-dependent [35]. However, a study on a kidney-transplanted pediatric population showed that treatment with a combination of steroids, calcineurin inhibitors, and antimetabolites, which is a common regimen for SLE, was not associated with an increase in CD57 expression [36]. In the present study, the patients’ cohort had relative variability in treatment regimen, with the majority receiving steroid treatment (90%) and only three patients receiving calcineurin inhibitors. Unfortunately, the sample size is too small to perform subgroup analysis, but we believe that further investigation is necessary to elucidate the degree to which these alterations were exacerbated by treatment factors. Furthermore, the inclusion of additional senescence- and exhaustion-related markers (such as KLRG-1 for senescent cells, CD108 for CD8 RTEs, PTK-7 for CD4 RTEs, and TIM-3, LAG3, TOX, and CD244 for exhaustion cells), along with a quantification of their expression by evaluating the mean fluorescence intensities in flow cytometry, would be useful in better defining the affected subsets, as well as their properties and engagement in LN physiopathology.

Overall, we investigated the alterations of a broad panel of senescence- and exhausted-related markers in LN patients and compared our findings to healthy controls. The LN patients were characterized by CD4+ T cells lymphopenia, affecting both naïve and memory subsets, with a preserved thymic function. However, in the CD8 compartment, naïve T cells were found to be severely decreased, with a concomitant increase in the memory subsets. Immune exhaustion affected both T cell populations; however, they were not affected in the same way. SLE is a chronic inflammatory condition, which is characterized by the presence of exhausted T cells and, to a lesser extent, the presence of senescent T cells subsets.

## 4. Materials and Methods

### 4.1. Patients

This is a cross-sectional study, in which the T lymphocytes immune phenotype of patients with LN was thoroughly investigated by analyzing the expression of lymphocyte surface molecules. The study was conducted in the department of Nephrology of Aristotle University of Thessaloniki, in collaboration with the Department of Immunology, National Peripheral Histocompatibility Center, Hippokration Hospital, Thessaloniki.

Patients included in the present study were adults diagnosed with SLE, based on the SLICC/ACR 2012 criteria [37], with renal involvement. Diagnosis of LN was based exclusively on kidney biopsy findings. At least one kidney biopsy was required for each patient, and the latest had to be performed no more than two years prior to their enrollment in the study. For patients who had more than one biopsy, the last one was used for evaluation. All patients require regular follow-ups, with available clinical, biochemical, and immunological data.

Patients with recent (<3 months) or active infection, recent (<6 months) administration of cyclophosphamide or monoclonal antibodies, active malignancy or history of malignant disease, diabetes mellitus, impaired renal function (defined as an estimated glomerulus filtration rate (eGFR) < 60 mL/min/m^2^), or patients who were uncompliant were excluded from the study.

The study was approved by the Institutional Review Board of the Medical School of Aristotle University of Thessaloniki, Greece. Both the patients and controls were informed about the study and signed the informed consent prior to enrollment.

### 4.2. Clinical Data and Renal Histology

The recorded clinical data included age at the time of diagnosis of the disease and at the time of the study, as well as disease duration and previous and current medication. Additionally, laboratory findings, such as hematological, biochemical, and immunological results were recorded at the time of evaluation. SLE activity was estimated based on the SLEDAI score [38].

Renal biopsies were re-assessed by a pathologist and a nephrologist, unaware of the clinical and laboratory findings, and classified according to the ISN/RPS 2003 system [39].

### 4.3. Flow Cytometry

Whole blood was drawn from the patients and controls under sterile conditions, collected in EDTA blood-collection tubes, and proceeded to the evaluation of total lymphocytes, proportions, and counts of the CD4 and CD8 subpopulations based on the expression of surface molecules. Flow cytometry was performed within less than 12 h after collection. The panel of surface molecules evaluated were decided according to their importance as markers of senescent and exhausted cells, as was described before [12]. Therefore, the expression of CD45RA, CCR7, CD28, CD31, CD57, and PD1 on both CD4 and CD8 lymphocytes was assessed using a cytometer (Navios Flow Cytometer, Beckman Coulter, Brea, CA, USA). Blood samples were stained with conjugated antibodies for anti-CD45 PC7 J33 (IM3548U, Beckman Coulter), anti-CD3 FITC UCHT1 (A07746, Beckman Coulter), anti-CD3 PE UCHT1 (A07747, Beckman Coulter), anti-CD4 Pacific blue MEM-241 (PB-359-T100, EXBIO, Praha SA), anti-CD8 PC5 B9.11 (A7758, Beckman Coulter), anti-CD45RA APC MEM-56 (1A-223-T100, EXBIO, Praha SA), anti-CCR7 PE 4B12 (1P-735-C100, EXBIO, Praha SA), anti-CD28 CD28.2 PE-EF610 (61-0289-42, ThermoScientific LSG), anti-CD31 APC MEM-05 (T5-273-T100, EXBIO, Praha SA), anti-CD57 FITC TB01 (1F-158-T100, EXBIO, Praha SA), and anti-CD279 (PD1) EI12.2H7 (11-176-C100, EXBIO, Praha SA). The combinations of conjugated antibodies used are described in Supplementary Table S1 and the gating strategy is shown in Supplementary Figures S1 and S2.

Based on the combined expression of the senescent-related markers, certain lymphocyte subpopulations were defined as naïve, memory, and senescent. Moreover, according to the co-expression of PD1 and CD45RA, we evaluated exhaustion on memory CD45RA− or EMRA T lymphocytes as presented in Supplementary Table S2. The absolute count of the lymphocyte subsets was calculated by the proportions from the cytometric data, and the count of lymphocytes was calculated from a routine whole-blood count from the same sample.

Twenty (20) healthy volunteers of similar race, age, and sex were used as the control group.

### 4.4. Statistics

Statistical analysis was performed using Statistical Package for Social Sciences (SPSS Inc., Chicago, IL, USA) software, version 27.0, for Windows.

Continuous variables were defined as having normal or non-normal distribution, based on the Shapiro–Wilk and Kolmogorov–Smirnov tests, and expressed as mean ± standard deviation (Mean ± Standard Deviation) or median and range [Medians (min-max)], respectively. An independent samples Student’s t test and Mann–Whitney U test, or Wilcoxon signed ranks test, were used to compare the differences between the continuous variables, while Pearson’s and Spearman’s correlation tests were applied to compare the variables with normal or non-normal distribution, respectively. The statistical significance threshold for all comparisons was set at *p* < 0.05.

## Figures and Tables

**Table 1:** Clinical and laboratory findings of patients with LN at time of diagnosis and time of evaluation (SLE: systematic lupus erythematosus, LN: Lupus Nephritis).

	At Diagnosis	At Evaluation
n	30	30
M/W	4/26	
Mean age at diagnosis (years)	31 ± 15	43 ± 16
SLE manifestations and Systems involved		
Skin and mucosal involvement	19/30 (63.3%)	13/30 (43.3%)
Musculoskeletal system	22/30 (73.3%)	7/30 (23.3%)
Respiratory system	3/30 (10%)	0/30 (0%)
Cardiovascular system	4/30 (13.3%)	1/30 (3.3%)
Hematological disorders	13/30 (43.3%)	3/30 (10%)
Kidney involvement	30/30 (100%)	22/30 (73.3%)
Neuropsychiatric disorders	3/30 (10%)	3/30 (10%)
Immunological/serological disorders	30/30 (100%)	16/30 (53.3%)
Mean SLEDAI-2K score		11
Renal Pathology Classification according to ISN/RPS 2003		
Stage II	3/30 (10%)	
Stage III	3/30 (10%)	
Stage IV	14/30 (46.7%)	
Stage V	7/30 (23.3%)	
Stage III + V	3/30 (10%)	

**Table 2:** Differences in percentages and absolute numbers (cells/μL) of CD4 lymphocyte subpopulations between LN patients and healthy controls (LN: lupus nephritis; RTEs: recent thymic emigrants; CM: central memory; EM: effector memory; EMRA: effector memory re-expressing CD45RA+; NS: non-significant).

	LN Patients	Healthy Controls	*p*
n	30	20	
Naïve CD4 lymphocytes			
CD4+CD31+ (%) (RTEs)	33.8 (12–49)	28.4 (6–60)	NS
CD4+CD31+	212 0–468)	279 (59–959)	NS
CD4+CD45RA+CCR7+ (%) (naïve)	38.3 (11–62)	46.7 (25–85)	NS
CD4+CD45RA+CCR7+	233.6 (0–572)	453 (231–1357)	0.02
CD4+CD28+CD57− (%)	94 (12–98)	96 (86–99)	NS
CD4+CD28+CD57−	550 (0–1228)	989 (664–1568)	<0.001
Memory CD4 lymphocytes			
CD4+CD45RA−CCR7+ (%) (CM)	55.8 (26–76)	48.3 (2.5–73)	NS
CD4+CD45RA−CCR7+	294 (0–972)	570 (39–1001)	0.009
CD4+CD45RA−CD57− (%)	53 (7–74)	52 (13–76)	NS
CD4+CD45RA−CD57-	236 (0–884)	605 (208–990)	<0.0001
CD4+CD45RA-CCR7− (%) (EM)	0.4 (0–23)	1 (0.1–4.5)	0.001
CD4+CD45RA−CCR7−	1.7 (0–73)	11 (1.5–43)	<0.001
CD4+CD28+CD57+ (%)	0.8 (0–86)	0.7 (0.3–1.7)	NS
CD4+CD28+CD57+	4.7 (0–806)	6.8 (3.8–23)	NS
CD4+CD45RA−CD57+ (%)	2 (0.3–43)	1.4 (0–5.5)	NS
CD4+CD45RA−CD57+	9.7 (0–408)	16.5 (0–73)	NS
Advanced Differentiated CD4 cells			
CD4+CD45RA+CCR7− (%) (EMRA)	1.7 (0–38)	2.5 (0.6–10)	NS
CD4+CD45RA+CCR7−	9.3 (0–123)	30 (5.9–167)	0.009
CD4+CD45RA+CCR7−CD28− (%) (EMRA-CD28−)	19.7 (0–100)	14 (1.9–28)	NS
CD4+CD45RA+CCR7−CD28− (EMRA-CD28−)	1.9 (0–18)	2.9 (0.3–18)	NS
CD4+CD28−CD57− (%)	2.2 (0–18)	1.1 (0.4–9)	NS
CD4+CD28−CD57−	7.2 (0–50)	12 (4–69)	NS
CD4+CD28−CD57+ (%)	1.7 (0.1–23)	2.2 (0.1–7.5)	NS
CD4+CD28−CD57+	9.9 (0–66)	25 (0.9–99)	NS
CD4+CD45RA+CD57+ (%)	1.3 (0–39)	1.6 (0.2–6.4)	NS
CD4+CD45RA+CD57+	6.7 (0–364)	14 (1.9–61)	NS

**Table 3:** Differences in percentages and absolute numbers (cells/μL) of CD8 lymphocyte subpopulations expressing markers of “aging” between patients with LN and healthy controls (LN: lupus nephritis, RTEs: recent thymic emigrants; CM: central memory; EM: effector memory; EMRA: effector memory re-expressing CD45RA+; NS: non-significant).

	LN Patients	Healthy Controls	*p*
n	30	20	
Naïve CD8 lymphocytes			
CD8+CD31+ (%) (RTEs)	33 (7.6–73)	40 (19–63)	NS
CD8+CD31+	84 (0–353)	207 (73–344)	0.01
CD8+CD45RA+CCR7+ (%) (naïve)	24 (0.8–72)	39 (21–95)	0.01
CD8+CD45RA+CCR7+	68 (0–347)	200 (82–804)	<0.001
CD8+CD45RA+CD57− (%)	27 (2.3–67)	37 (12–77)	NS
CD8+CD45RA+CD57−	58 (0–324)	176 (68–402)	0.003
CD8+CD28+CD57− (%)	55.8 (3.3–85)	63 (25–82)	NS
CD8+CD28+CD57−	168 (0–373)	318 (153–540)	0.002
Memory CD8 lymphocytes			
CD8+CD45RA−CCR7+ (%) (CM)	39 (1.8–88)	15 (0.1–55)	0.001
CD8+CD45RA−CCR7+	100 (0–472)	60 (0.6–220)	NS
CD8+CD45RA−CD57− (%)	38 (1.1–84)	39 (5.5–65)	NS
CD8+CD45RA−CD57−	117 (0–401)	134 (28–386)	NS
CD8+CD45RA−CCR7− (%) (EM)	4.9 (0.4–55)	1.2 (0–27)	NS
CD8+CD45RA+CCR7−	13.9 (0–97)	2.4 (0–255)	0.03
CD8+CD28+CD57+ (%)	1.9 (0.5–79)	1.8 (0.5–4.7)	NS
CD8+CD28+CD57+	7.4 (0–132)	10 (1.4–37)	NS
CD8+CD45RA−CD57+ (%)	12.8 (1.2–52)	9.1 (0.6–24)	NS
CD8+CD45RA−CD57+	25.5 (0–277)	55.8 (2–118)	NS
Advanced Differentiated CD8 cells			
CD8+CD45RA+CCR7− (%) (EMRA)	8.9 (0–58)	28.3 (2.9–67)	NS
CD8+CD45RA+CCR7−	19 (0–279)	143 (6.2–463)	0.008
CD8+CD45RA+CCR7−CD28− (%) (EMRA-CD28−)	33 (5.6–99)	40 (10–71)	NS
CD8+CD45RA+CCR7−CD28− (EMRA-CD28−)	5.7 (0–192)	35.8 (2.5–221)	0.004
CD8+CD28−CD57− (%)	13 (0.2–36)	10.8 (4–19)	NS
CD8+CD28−CD57−	43 (0–194)	49 (27–126)	NS
CD8+CD28−CD57+ (%)	19 (2.2–59)	26 (4.2–66)	NS
CD8+CD28−CD57+	53 (0–344)	97 (19–432)	NS
CD8+CD45RA+CD57+ (%)	5.7 (0.3–49)	12.3 (2–50)	NS
CD8+CD45RA+CD57+	15.8 (0–178)	47 (8.7–327)	0.01

**Table 4:** Expression of PD1 molecule on CD4 and CD8 lymphocytes, differences between LN patients and HCs (LN: lupus nephritis, NS: non-significant).

	LN Patients	Healthy Controls	*p*
n	30	20	
CD4+PD1+ (%)	11.6 (3.4–97.9)	8.2 (1.6–16.2)	0.03
CD4+PD1+	65.6 (0–606.9)	78.5 (15.3–189.4)	NS
CD4+CD45RA+PD1+ (%)	2.3 (0.5–49)	0.6 (0–1.7)	<0.0001
CD4+CD45RA+PD1+	11.7 (0–291)	6.7 (0–21)	0.03
CD4+CD45RA−PD1+ (%)	10 (2.8–67)	7.7 (1.5–15)	NS
CD4+CD45RA−PD1+	57 (0–358)	73 (14–180)	NS
CD8+PD1+ (%)	28.8 (9.7–96.9)	12.5 (4–33.4)	0.001
CD8+PD1+	81.5 (0–425)	46.4 (18–142.3)	NS
CD8+CD45RA+PD1+ (%)	11 (0.4–63)	3.6 (1.2–10)	0.01
CD8+CD45RA+PD1+	24 (0–305)	18 (5.4–54)	NS
CD8+CD45RA−PD1+ (%)	18.6 (7–88)	9 (0.7–27)	0.008
CD8+CD45RA−PD1+	65.8 (0–274)	31.5 (3–125)	NS

**Table 5:** Differences in immunophenotype of CD4 lymphocytes in relation to histological classification (RTEs: recent thymic emigrants; CM: central memory; EM: effector memory; EMRA: effector memory re-expressing CD45RA+; NS: non-significant).

	Stage II	Stage III	Stage IV	Stage V	*p*	*p* (II/V vs. III/IV)
CD4 Lymphocytes and Subtypes	n = 2	n = 4	n = 9	n = 5		
CD4 (%)	44.9 (9.1)	59.9 (20.7)	45.4 (19.7)	58.1 (15.5)	0.04	NS
CD4 (cells/μL)	539.4 (109)	621.5 (652.5)	378.1 (590.4)	941.9 (414.3)	NS	NS
CD4+CD31+ (RTEs) (%)	40 (0.4)	33 (8.7)	21.5 (14.8)	38 (10.4)	0.02	0.002
CD4+CD45RA+CCR7+ (naïve) (%)	55.2 (14.1)	38.3 (21.2)	30.4 (15.6)	45.7 (7.3)	NS	0.01
CD4+CD45RA−CCR7+ (CM) (%)	39.6 (4.1)	59.4 (22.6)	66.8 (24.2)	51.1 (10.8)	NS	0.03
CD4+CD45RA−CCR7− (EM) (%)	0.9 (1.9)	0.3 (0.7)	0.5 (0.6)	0.4 (0.7)	NS	NS
CD4+CD45RA+CCR7− (EMRA) (%)	4.4 (8.8)	1.7 (1.2)	1.7 (3.2)	2.9 (3.2)	NS	NS
CD4+CD45RA+CCR7-CD28− (CD28-EMRA) (%)	13.6 (27.2)	15.3 (36.3)	23.7 (20)	8.5 (5.1)	NS	<0.001
CD4+CD28+CD57+ (%)	0.9 (0.2)	1 (2.8)	0.7 (0.6)	1 (64.4)	NS	NS
CD4+CD28+CD57− (%)	96.1 (5.6)	92.4 (11.5)	93.2 (12.1)	97 (65.2)	NS	NS
CD4+CD28−CD57− (%)	1.1 (1.9)	0.9 (2.6)	3.6 (5.1)	0.6 (2.2)	0.04	0.02
CD4+CD28−CD57+ (%)	1.8 (3.5)	3.2 (11.3)	2.3 (6.9)	0.2 (0.8)	NS	NS
CD4+CD45+CD57− (%)	58,3 (11.8)	38,8 (17,7)	32 (31,6)	48,9 (40,9)	NS	NS
CD4+CD45−CD57− (%)	38.9 (7.3)	57 (22.4)	58.2 (26.1)	41.5 (37.2)	NS	0.01
CD4+CD45−CD57+ (%)	1 (1.3)	2.6 (3.5)	3.3 (11.3)	0.7 (32.6)	NS	NS
CD4+CD45+CD57+ (%)	1.7 (3.2)	3.6 (7.4)	1.6 (4.9)	0.5 (28.9)	NS	NS

## Data Availability

Data are available upon request.

## Supplementary Materials

The following supporting information can be downloaded at: https://www.mdpi.com/article/10.3390/ijms232213928/s1.
