# Supplementary material for: Exhausted but Not Senescent T Lymphocytes Predominate in Lupus Nephritis Patients
Source: Int J Mol Sci. 2022 Nov 11;23(22):13928. doi: 10.3390/ijms232213928 (PMC9694088; doi:10.3390/ijms232213928)
Supplement: Supplementary file 1 [file ijms-23-13928-s001.zip › ijms-1996487-supplementary.pdf]

Supplement S1

Flow Cytometry

Blood samples were stained with conjugated antibodies for anti-CD45 PC7 IM3548U (Beckman Coulter), anti-CD3 FITC UCHT1 (Beckman Coulter), anti-CD3 PE UCHT1 (Beckman Coulter), anti-CD4 Pacific blue EM4 (EXBIO, Praha SA), anti-CD8 PC5 (IM3548U -Beckman Coulter), anti-CD45RA APC MEM-56 (EXBIO, Praha SA), anti-CCR7 PE 4B12 (EXBIO, Praha SA), anti-CD28 CD28.2 PE-EF610 (ThermoScientific LSG), anti-CD31 APC MEM-05 (EXBIO, Praha SA) and anti-CD57 FITC (EXBIO, Praha SA). CD4 and CD8 lymphocyte subsets were determined using a cell counter (Navios Flow Cytometer, Beckman Coulter), according to the manufacturer's recommendations. Three antibody panels were used for each sample as shown in the Table S1.

Table S1. T lymphocyte surface markers combinations

**Tube 1**	**Tube 2**	**Tube 3**
CD45 PC7	CD45 PC7	CD45 PC7
CD3 FITC	CD3 PE	CD3 FITC
CD4 Pacific Blue	CD4 Pacific Blue	CD4 Pacific Blue
CD8 PC5	CD8 PC5	CD8 PC5
CD45RA APC	CD45RA APC	CD45RA APC
CCR7 PE	CD57 FITC	CD31 ECD
CD28 ECD	CD28 ECD	PD1 PE

Table S2. Expression of T lymphocyte surface molecules on different CD4 and CD8 T cells subsets, and phenotype of subsets determined (RTEs: recent thymic emigrants, CM: central memory, EM: effector memory, EMRA: effector memory re-expressing CD45RA)

**T lymphocytes markers**			
	**Naive lymphocytes**	**Memory lymphocytes**	**Senescent lymphocytes**
**CD31**	+	-	-
**CD45RA**	+	-	+
**CCR7**	+	±	-
**CD57**	-	±	+
**CD28**	+	±	-
**PD-1**	-	±	±
**T lymphocyte phenotypes**			
	**Naive lymphocytes**	**Memory lymphocytes**	**Senescent lymphocytes**
**CD4 cells**	CD4+CD31+ (RTEs)	CD4+CD45RA-CCR7+ (CM)	CD4+CD45RA+CCR7-
	CD4+CD45RA+CCR7+	CD4+CD45RA-CD57-	CD4+CD45RA+CCR7-CD28- (EMRA-CD28null)
	CD4+CD28+CD57-	CD4+CD45RA-CCR7- (EM)	CD4+CD28-CD57-
		CD4+CD28+CD57+	CD4+CD28-CD57+
		CD4+CD45RA-CD57+	CD4+CD45RA+CD57+
		CD4+PD1+CD45RA-	CD4+PD1+CD45RA+
**CD8 cells**	CD8+CD31+ (RTEs)	CD8+CD45RA-CCR7+ (CM)	CD8+CD45RA+CCR7-
	CD8+CD45RA+CCR7+	CD8+CD45RA-CD57-	CD8+CD45RA+CCR7-CD28- (EMRA-CD28null)
	CD8+CD28+CD57-	CD8+CD45RA-CCR7- (EM)	CD8+CD28-CD57-
		CD8+CD28+CD57+	CD8+CD28-CD57+
		CD8+CD45RA-CD57+	CD8+CD45RA+CD57+
		CD8+PD1+CD45RA-	CD8+PD1+CD45RA+

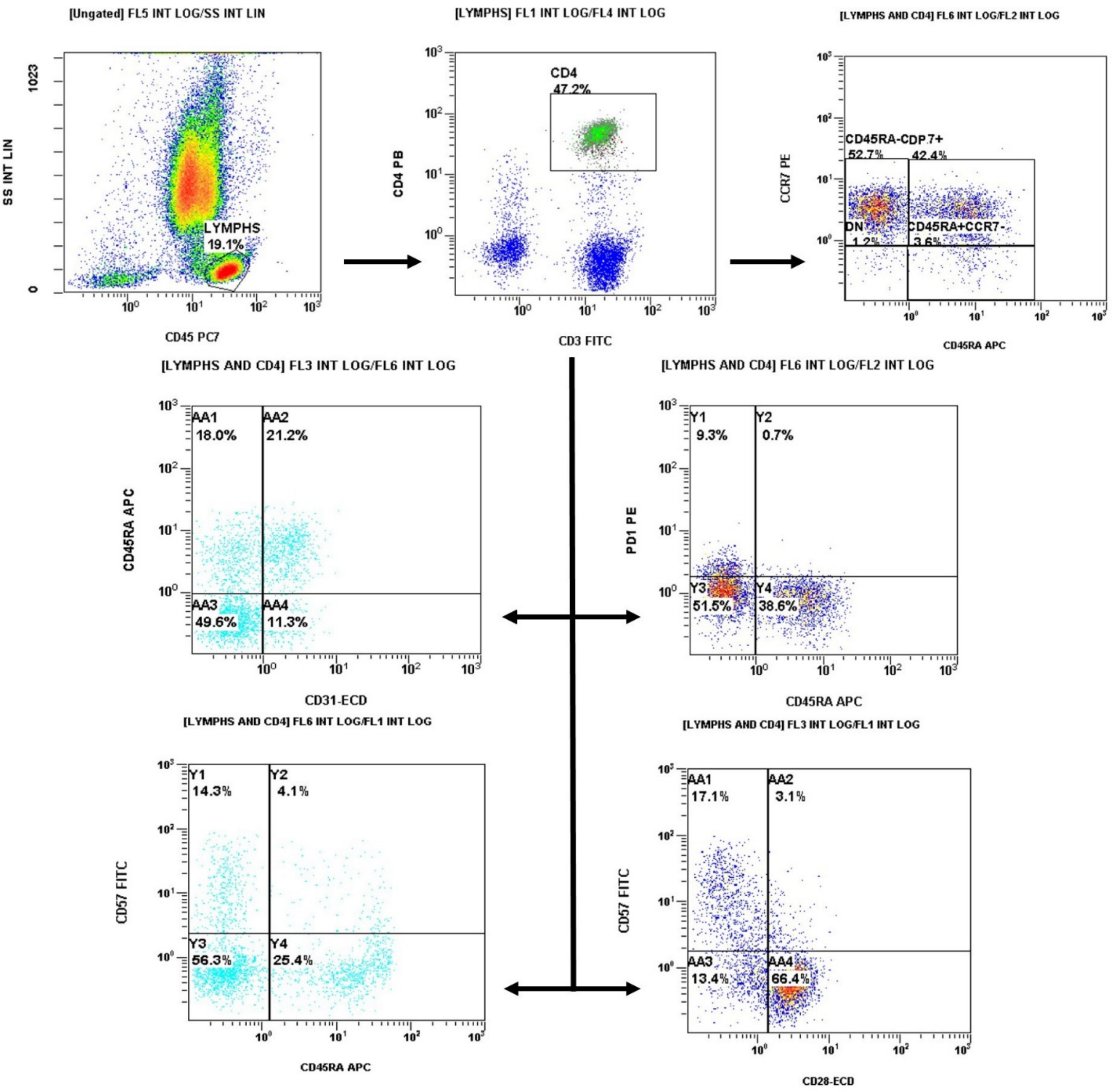

Figure S1. Gating strategy for CD4+ lymphocytes.

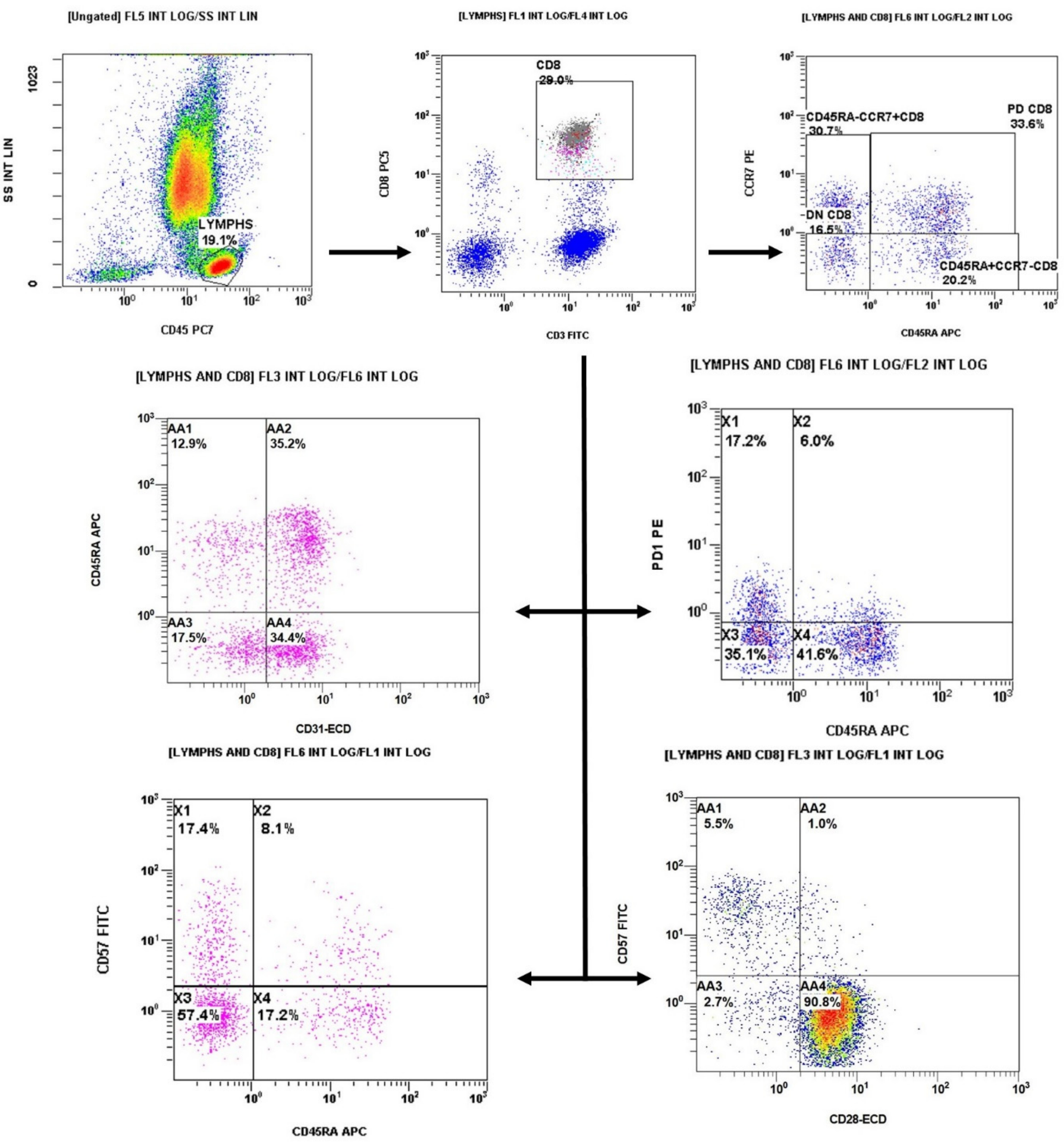

Figure S2. Gating strategy for CD8+ lymphocytes.
